# Optimizing the Management and Outcomes of Failed Back Surgery Syndrome: A Proposal of a Standardized Multidisciplinary Team Care Pathway

**DOI:** 10.1155/2019/8184592

**Published:** 2019-07-08

**Authors:** Kliment Gatzinsky, Sam Eldabe, Jean-Philippe Deneuville, Wim Duyvendak, Nicolas Naiditch, Jean-Pierre Van Buyten, Philippe Rigoard

**Affiliations:** ^1^Department of Neurosurgery, Sahlgrenska University Hospital, Gothenburg, Sweden; ^2^Department of Pain and Anaesthesia, The James Cook University Hospital, Middlesbrough, UK; ^3^Spine & Neuromodulation Functional Unit, Poitiers University Hospital, Poitiers, France; ^4^Institut Pprime UPR 3346, CNRS, University of Poitiers, Poitiers, ISAE-ENSMA, France; ^5^PRISMATICS Lab (Predictive Research in Spine/Neuromodulation Management and Thoracic Innovation/Cardiac Surgery), Poitiers University Hospital, Poitiers, France; ^6^Department of Neurosurgery, Jessa Hospital, Hasselt, Belgium; ^7^Department of Anesthesia and Pain Management, Hospital AZ Nikolaas, Sint-Niklaas, Belgium

## Abstract

Failed back surgery syndrome (FBSS) is a major, worldwide health problem that generates considerable expense for healthcare systems. A number of controversial issues concerning the management of FBSS are regularly debated, but no clear consensus has been reached. This pitfall is the result of lack of a standardized care pathway due to insufficient characterization of underlying pathophysiological mechanisms, which are essential to identify in order to offer appropriate treatment, and the paucity of evidence of treatment outcomes. In an attempt to address the challenges and barriers in the clinical management of FBSS, an international panel of physicians with a special interest in FBSS established the Chronic Back and Leg Pain (CBLP) Network with the primary intention to provide recommendations through consensus on how to optimize outcomes. In the first of a series of two papers, a definition of FBSS was delineated with specification of criteria for patient assessment and identification of appropriate evaluation tools in order to choose the right treatment options. In this second paper, we present a proposal of a standardized care pathway aiming to guide clinicians in their decision-making on how to optimize their management of FBSS patients. The utilization of a multidisciplinary approach is emphasized to ensure that care is provided in a uniform manner to reduce variation in practice and improve patient outcomes.

## 1. Introduction

A significant proportion of patients who have undergone lumbar spinal surgery continue to suffer from persistent pain and impaired function, referred to as failed back surgery syndrome (FBSS) [1–5]. Patients with FBSS are a heterogeneous group, with complex and varied aetiologies, and typically present with chronic back or extremity pain, often both [1]. They have a low health-related quality of life (HRQoL) and high psychological morbidity and are frequent users of health services [2–4].

Failed back surgery syndrome is a condition that is difficult to treat successfully because of (a) lack of a precise pathophysiology and complexity of presentation [4, 6–10], (b) lack of a gold standard therapy or one-size-fits-all solution [11], and (c) limited availability of clinical guidance [12]. Patients with FBSS are at risk of being confined to the care of a single discipline, and treatment recommendations are often determined by the managing healthcare provider's experience [13]. Although repeat surgery has been shown to be less successful than the primary surgery in several studies [14–18], awareness of available, alternative treatment options is often limited among surgeons, which may lead to further treatment delay and economic inefficiencies.

There is a growing trend towards evidence-based medicine that requires clinical decisions to be based on well-documented results taking the patient's best interests and the pain physician's/surgeon's experience into account. While this approach has been very successful in other fields of medicine, limited data are available concerning many issues related to the management of FBSS despite new validated therapeutic options. This lack of good quality data not only makes it difficult to utilize an evidence-based paradigm in the routine management of FBSS but also makes the optimal choice of treatment options for patients difficult.

The complexity of FBSS suggests that a multidisciplinary team (MDT) approach is important for the optimization of outcomes [19–22]. However, the management of patients with FBSS is often complicated by limited access to specialist pain centers offering the clinical expertise of multiple professional disciplines. While there are some published treatment pathways and algorithms following this main principle, there is no standardized care pathway for FBSS based on an MDT approach to provide guidance on assessment, treatment, and long-term evaluation of patients with FBSS to clinicians in order to optimize treatment outcomes.

To address the challenges of defining a comprehensive FBSS care pathway, an international panel of physicians with a special interest in FBSS established the Chronic Back and Leg Pain (CBLP) Network with the goal to provide recommendations on the management of patients with FBSS based on a multidisciplinary input. The work is presented in a series of two papers. The first paper focused on the definition of FBSS and outlined the criteria for appropriate diagnosis, with recommendations of validated tools to improve patient assessment [23]. The goal of this paper is to present a standardized care pathway to support clinicians in their decision-making on how to assess, treat, and evaluate patients with FBSS from an MDT-based perspective.

## 2. Materials and Methods

### 2.1. The Chronic Back and Leg Pain Network Constitution and Methodology

**Figure 1:**
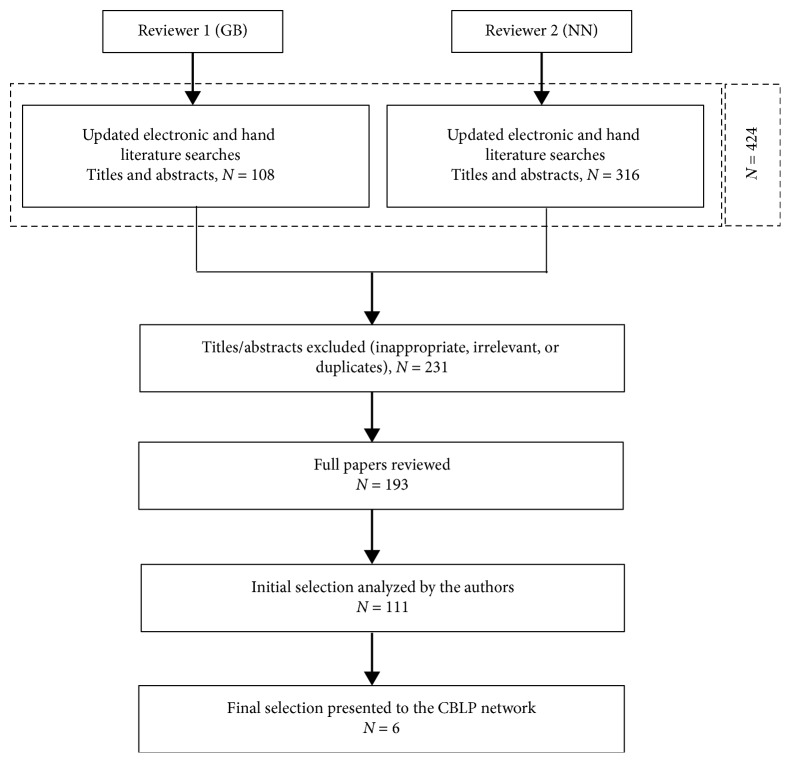
Diagram summarizing literature searches: FBSS management. The electronic and hand literature searches yielded 424 titles. Following a review of full-text versions of the 177 (NN) + 16 (GB) residual publications, after discarding duplicates and initial exclusion of 231 titles/abstracts, 95 (NN) + 16 (GB) papers were finally selected and 6 were retained. These are presented in Table 1.

The composition of the CBLP Network and the methodology used to develop the proposed standardized FBSS care pathway adhere to the outlines presented in our first paper on FBSS definition and guidelines for patient assessment [23]:

- Participants in the CBLP panel were selected based on their extensive clinical and scientific experience in managing FBSS patients with focus on representation of the three specialties that are most involved in the treatment of this patient population: orthopaedic surgery, neurosurgery, and pain medicine/anesthesiology. Invitations were sent to potential participants all over Europe and accepted prior to engagement in the panel. Formal face-to-face meetings were held on a regular basis from 2012 to 2016 with additional follow-up teleconferences. All meetings were chaired by a trained facilitator to help the consensus process. Additional input was provided on an ongoing basis by relevant clinical specialists involved in the multidisciplinary evaluation and treatment of patients with FBSS (psychologist, psychiatrist, physiotherapist, and rehabilitation physician).
- Systematic literature searches in PubMed, MEDLINE, LILACS, Embase, and the National Guideline Clearinghouse were conducted by two separate reviewers (one independent reviewer = GB and one reviewer on behalf of the group = NN) on a regular basis up to September 2018, without any restrictions regarding the language or year of publication. The search strategy was developed in order to maximize sensitivity of article identification, using controlled vocabulary and title/abstract words combining variations of “Failed back surgery syndrome,” “Back pain,” “Chronic leg pain” with “Multidisciplinary” OR “Team,” “Clinical pathway” OR “Practice guideline” OR “Algorithm” OR “Guideline” OR “Protocol” (detailed description hereafter). The literature searches in this paper focus on therapeutic strategies and algorithms. For the independent reviewer (GB), the term “Failed back surgery syndrome” was cross-referenced with terms pertaining to clinical guidelines or algorithms (i.e., “Clinical pathway” OR “Practice guideline” OR “Algorithm”). Hand-searching of reference lists of identified reports and relevant review articles was also carried out. For the group reviewer (NN), the search strategy varied according to the database as follows:
  - MEDLINE: (“Failed back surgery syndrome” OR “Chronic Back pain” OR “Chronic leg pain”) AND (“Multidisciplinary” OR “Interdisciplinary” OR “Team” OR “Clinical pathway” OR “Guideline” OR “Protocol” OR “Algorithm”)
  - LILACS: (“Failed back surgery syndrome”) AND (“Multidisciplinary” OR “Interdisciplinary” OR “Team” OR “Pathway” OR “Guideline” OR “Protocol” OR “Algorithm”)
- All references retrieved from databases were exported to Zotero to identify and exclude duplicated studies.
- The two literature searches were pooled and crossed to converge into one final diagram. Our methodology is summarized in Figure 1.
- The final literature review ensured that the CBLP Network members had access to the same body of evidence during the panel discussions.
- Consensus was defined as full agreement on the set goals which was achieved during the facilitated round table discussions, based on the outcomes of the literature overview, each member's personal experience, and the additional input from relevant clinical specialists. The consensus process did not include any individual (independent and anonymous) rating rounds based on the Delphi method since the number of participants was considered to be too small and the purpose of the discussions was not to measure consensus based on specific statements, but to resolve disagreements (reach full consensus) on the set task [27]. The limitations of the chosen methodology to reach consensus are discussed in our first paper on FBSS definition and patient assessment [23].

## 3. Results

### 3.1. Proposed Care Pathway in the Management of FBSS Utilizing an MDT

Six comprehensive FBSS care pathways or algorithms were identified by the literature searches (Table 1) [2, 12, 20, 24–26]. Their successful application and adoption in clinical practice have, however, been constrained by focusing on one treatment or investigation of a single FBSS subgroup, with lack of standardization. None of the algorithms met all of the following criteria for the development of an algorithm or care pathway: focus on all available aspects and means for patient evaluation and therapeutic options, emphasis on the involvement of an MDT, and evidence that a wide variety of experts provided consensus in its development.

**Figure 2:**
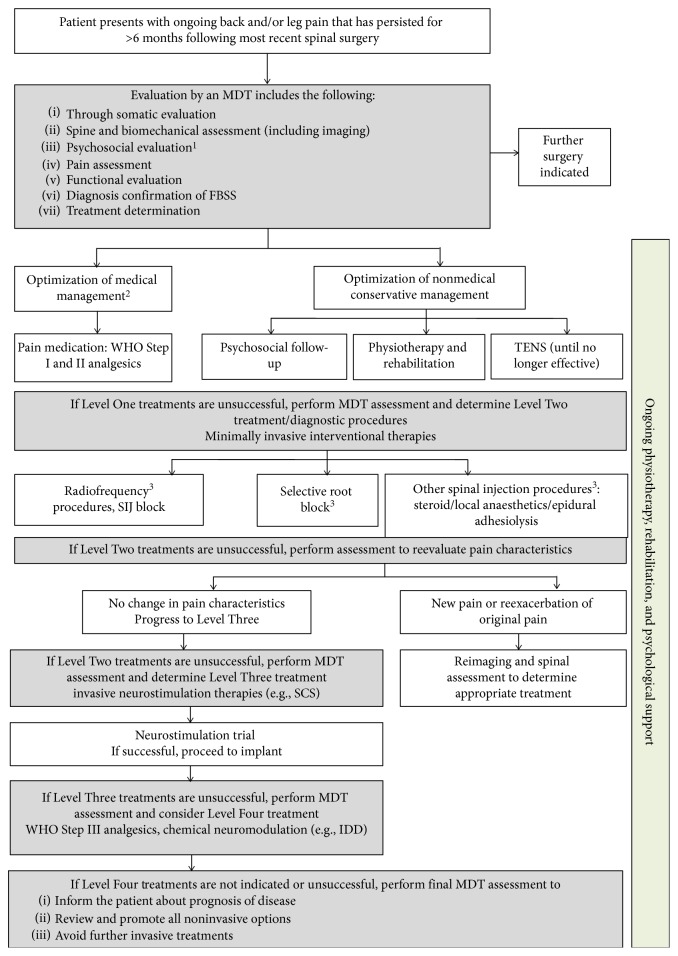
The proposed standardized multidisciplinary team's failed back surgery syndrome care pathway, as recommended by the Chronic Back and Leg Pain Network. FBSS, failed back surgery syndrome; IDD, intrathecal drug delivery; MDT, multidisciplinary team; SCS, spinal cord stimulation; SIJ, sacroiliac joint; TENS, transcutaneous electrical nerve stimulation; WHO, World Health Organization. *Note.* In cases of new pain and/or exacerbation of original pain at any stage of this flow, reimaging and spine expertise is required. ^1^Best practice is for the psychosocial evaluation to be performed by a psychologist or psychiatrist with specific experience in the field of pain. Assessments may include the relevant tests and questionnaires aiming to identify patients with major psychological or psychiatric contraindications [23]. ^2^Best practice is to avoid long-term use of WHO Step III analgesics and review ineffective long-term use of antineuropathic pain medication [28–30]. ^3^There is limited evidence supporting a prolonged effect of epidural injections, selective nerve root blocks, and radiofrequency denervation in an FBSS population [20, 25, 31, 32]. Despite this lack of clinical evidence, these therapies may be tried/reserved for the management of acute exacerbation in pain.

In response to the identified limitations of current practice in the caretaking of FBSS patients, the CBLP Network developed a care pathway based on an MDT input to serve as a quick reference decision resource (Figure 2). The pathway focuses equally on (1) appropriate clinical evaluation for adequate patient selection and (2) elucidation of the full range of available treatments and diagnostic procedures and their place in the overall continuum of care using an evidence-based approach, as summarized in Figure 2.

### 3.2. Level One Treatment

If a specific spinal aetiology for pain has been identified without demonstrating the need for further surgery and significant psychosocial comorbidities have been ruled out, Level One treatment can be initiated (Figure 2). The goal of the first-line therapy is to optimize nonmedical and medical, conservative management [20].

Consideration should at first hand be given to physiotherapy, rehabilitation, and management of psychological and social factors [33]. It is important to note that even though many clinical trials using these modalities to relieve pain have been conducted, their clinical effects on FBSS remain inconclusive [5, 34]. There is, however, growing evidence showing that a structured, mixed rehabilitative approach [35] combining pain education [36, 37], behavioral approach [38], and patient-centered exercise programs aiming to gradually expose the patient to fearful or painful movement to improve function [39] seems more effective than traditional rehabilitation programs [40].

At this stage in the care continuum, pharmacological therapy traditionally includes the World Health Organization (WHO) Step I and II analgesics only, preferentially utilizing nonnarcotic medication, such as nonsteroid anti-inflammatory drugs (NSAIDs) and paracetamol for treatment of pain of nociceptive origin [28]. Adjuvant short-term therapy with weak Step II opioids, e.g., tramadol or combinations of paracetamol and codeine, can be added to enhance the effects of nonopioid analgesics [33]. Given the lack of evidence of long-term effectiveness and clear evidence of harm associated with long-term use, WHO Step III analgesics with strong opioids should be avoided [41].

The pharmacological treatment of FBSS with a predominant neuropathic radicular component is based on the use of gabapentinoids (gabapentin and pregabalin) and antidepressants (amitriptyline and duloxetine) [20]. Two-drug combinations for the treatment of neuropathic pain in adults have been shown to improve analgesic efficacy [42]. Attention should, however, be payed to the potential risk of gabapentinoid dependency and abuse [43]. New data indicate that combinations of gabapentinoids and opioids are associated with an increased risk of opioid-related death [44]. The UK regulator recently reclassified gabapentinoids as Class C controlled drugs [45]. Furthermore, the effect of pregabalin and gabapentin in reducing the neuropathic leg pain in patients, including those with FBSS, has also been questioned [46, 47]. Hence, the use of gabapentinoid medication in the long term should be carefully reviewed [48].

The patient should be prescribed at least two different drugs consecutively for six weeks or more to determine treatment effects. If therapy is effective (at least a 30% improvement) [49], the first-line option is continued until deterioration is reported. If deterioration occurs or pain is refractory to treatment, second-line therapy options should be considered.

Transcutaneous electrical nerve stimulation may provide an alternative/complement to medication in patients with FBSS. Its effectiveness in chronic low back pain is, however, still controversial [50, 51]. Other nonpharmacological complementary therapies, such as acupuncture, manual therapy, functional restoration, and cognitive behavioral therapy, may also be utilized, although the level of evidence supporting most of these therapies in the management of chronic back pain is moderate at best [52, 53].

### 3.3. Level Two Treatment

Level Two treatment includes minimally invasive interventional therapies/diagnostic procedures. Several reviews and evidence-based clinical practice guidelines and recommendations for interventional techniques in the diagnosis and treatment of chronic spinal pain, including FBSS, have been published and may be consulted for guidance [54–56]. Before second-line treatment is initiated, the patient should be reassessed. If a nociceptive pain component remains clinically significant, either in the back or in the leg or in both, a differential interventional strategy to clearly identify the potential pain generator will be a prerequisite to choose the best interventional option [57]. For example, if the sacroiliac joint (SIJ) is suspected to be a significant potential nociceptive pain generator, but a combination of clinical tests is negative (negative likelihood ratio (LR) −0.11), the posttest probability for SIJ pain is low. When this combination is positive (LR +7.0), SIJ could be the source of nociception [57]. These clinical findings need to be confirmed by a reference standard procedure, which in this case is an SIJ double anaesthetic block [58]. If a double-block procedure confirms the SIJ as a source of nociception, treatments such as steroid injections or radiofrequency ablation can be administered [59, 60]. The same approach can be used for other potential spine pain generators, such as lumbar facet pain [57, 61–64].

Second-line procedures also include selective nerve root block injections for neuropathic pain. If successful, pulsed radiofrequency or spinal cord stimulation (SCS) (see Level Three Treatment) may be considered to achieve a more sustained effect [19, 65]. Practitioners should be mindful of the paucity of evidence for the long-term effects of pulsed radiofrequency procedures and spinal injections on the FBSS population [20, 25, 31, 32, 34]. Despite the lack of robust clinical evidence, it is the view of the authors that these therapies may be useful for the management of acute exacerbation of pain, with the awareness of disappointing results in the long term.

Several systematic reviews have demonstrated sustained pain relief (up to 24 months) with percutaneous epidural adhesiolysis in the management of FBSS due to epidural/perineural fibrosis or scarring as the anticipated pain generator [34, 66–68]. Epidural adhesiolysis may be used when other less invasive Level Two treatment modalities have been ineffective. The procedure requires special technical skills and is considered to be of low risk for serious adverse events when performed by well-trained physicians.

### 3.4. Level Three Treatment

Level Three treatment includes interventional electrical neurostimulation therapies which mainly target the neuropathic pain component in FBSS. Before third-line treatment with neurostimulation is initiated, the patient should be assessed by an MDT to determine eligibility. Spinal cord stimulation (SCS) is the most commonly used interventional neurostimulation treatment for refractory chronic pain, the beneficial effects of which may persist for many years [5, 19, 34, 69, 70]. Spinal cord stimulation is a safe therapy because it is a minimally invasive and reversible procedure with exceedingly few serious complications [71–73]. Randomized controlled trials have demonstrated favourable long-term outcomes of SCS compared with conventional medical management [74, 75] and reoperation [76] in treating the radicular, neuropathic leg pain component in FBSS. Spinal cord stimulation is effective in reducing pain and medication use and improving HRQoL, function, and sleep in this subset of FBSS patients [74, 76–78]. The back pain component, on the contrary, has posed a major treatment challenge. Several new treatment options, involving refinement of traditional paraesthesia-based SCS, have evolved in order to find a solution to this problem. Recent reports on the use of multicolumn leads utilizing an algorithmic programming approach [79–81], peripheral nerve field stimulation, either alone or in combination with SCS [82–86], 1–10 kHz high-frequency stimulation [87–89], so-called burst stimulation [90], and closed-loop stimulation [91] have presented with varying success in treating the axial low back pain component in FBSS patients.

### 3.5. Level Four Treatment

For a patient whose pain is not sufficiently controlled by or who is ineligible for minimally invasive interventional pain management techniques, WHO Step III analgesic pain medication with strong oral opioids may be prescribed and monitored until the patient experiences intolerable drug-related adverse events or fails to achieve the primary aim of improvement in function because of development of tolerance or hyperalgesia. This approach is controversial and has been subject to intense debate during the last years since high-dose medication with potent opioids is often associated with severe side effects, such as hormonal dysfunction, weight gain, constipation, hyperalgesia, development of tolerance with time and the potential for dependence, abuse and addiction, and death by overdose [92–95]. In addition to the side effects, outcome data examining the long-term efficacy of opioids in treatment of FBSS-related chronic pain are lacking [29]. In a recent RCT with masked outcome assessment, it was shown that treatment with opioids was not superior to treatment with nonopioid medications for improving pain-related functions over 12 months in patients with chronic back pain [96]. Returning to work has also been shown to be negatively associated with chronic opioid therapy in patients with persistent pain after lumbar fusion surgery for degenerative disc disease [97]. It is widely accepted that the use of high-potency opioids should be limited in the treatment of chronic pain.

Before initiating Level Four treatment, predictors of risk of long-term opioid use, such as duration of opioid intake in the year before lumbar surgery, refusion surgery, and any diagnosis of depression, have to be identified [30]. Patients prescribed WHO Step III analgesics should be followed up by the MDT at least three times per year to avoid uncontrolled increase in daily dose.

Chemical neuromodulation by continuous intrathecal drug delivery (IDD) based on morphine or ziconotide administration may be considered for patients preferentially with neuropathic pain who have responded to strong oral opioids in the presence of severe adverse events [98–100]. No protocols have specifically been developed for FBSS in this context. Long-term intrathecal opioid administration is associated with an increased risk of late respiratory distress and pronounced side effects on hormone levels [100]. In addition, there is lack of prospective randomized, placebo-controlled studies to ratify the effect of IDD on treatment of FBSS in the long term.

If Level Four treatments are unsuccessful, a final MDT assessment is performed to inform the patient about the prognosis of the disease, to motivate the need of avoiding further invasive treatments, and to review and promote all available noninvasive options.

The CBLP Network recommends that structured physical and rehabilitation therapy and psychological support are provided on an ongoing basis and that a patient's disability/function and HRQoL are reevaluated before each new line of therapy using the same instruments as those administered before treatment for FBSS was initiated [101]. In cases of new pain and/or exacerbation of original pain at any stage, reevaluation as well as reimaging and new spine expertise is required in order to exclude indication for further surgery.

## 4. Discussion

Clinical guidelines and treatment algorithms have increased in popularity in disease management in an era of rising healthcare costs. Because of higher demands for efficient care, the availability of costly technologies, variations in service delivery among and between providers, and the overuse of inappropriate services and therapies, clinicians, payers, and policy-makers view such decision tools as instruments that can make healthcare delivery more efficient and consistent [102, 103].

Failed back surgery syndrome remains difficult to treat successfully not only because of the lack of a precise pathophysiology and complexity of its clinical presentation [4, 6–10] but also because of the lack of a gold standard therapy or one-size-fits-all solution [11] and the limited availability of clinical guidance [12]. There is a consensus in the literature, as well as among members of the CBLP Network, that patients with FBSS are at risk of being confined to the care of a single discipline and that differences in treatment recommendations are often determined by the managing healthcare provider's experience and discipline [3, 13, 23].

The development of the care pathway presented by the CBLP Network has been driven by an interest in and understanding the role and application of the available treatment options for FBSS in real-life practice, particularly in view of the recent reports of higher harm rates and inefficacy of opioids and gabapentinoids in treatment of chronic nonmalignant pain. Compared to previously published FBSS care pathways and algorithms, the CBLP Network's pathway puts an emphasis on amalgamation of three main criteria to further improve the quality and reliability of the pathway and to facilitate its adoption into clinical practice. The three cornerstones are (i) focus on all available aspects and means for patient evaluation and optimal utilization of therapeutic options, (ii) emphasis on the involvement of an MDT to improve decision-making, and (iii) involvement of a wide variety of experts who provide consensus in the development of the pathway. A quick reference care pathway for the assessment, treatment, and evaluation of outcomes with an integrated multidisciplinary approach is an important resource for specialist and nonspecialist clinicians who manage patients with FBSS [19–22].

One major challenge in the development of the presented care pathway was that the evidence of the clinical outcomes in the FBSS population has not been clearly determined in the available literature, even though a multitude of clinical trials using different therapeutic approaches with the intention of relieving pain and improving function have been conducted. Only a few studies have systematically analyzed and evaluated the overall clinical trial data using an evidence-based approach. Because of the paucity of evidence-based guidelines in the management of FBSS, the CBLP Network chose to adhere to a consensus-based approach to achieve the set goals to define FBSS and design outlines for appropriate patient evaluation and to propose a concise treatment pathway. Limitations of the used approach are discussed in the first of the two papers in this series on how to optimize outcomes of FBSS [23].

In a recent systematic review, the literature on various modalities for treating the back pain and/or radiating leg pain component in FBSS was critically analyzed by means of quality assessment and level of evidence for each modality [34]. The review established that, among the many treatment options that have been outlined in the care pathway developed by the CBLP Network, epidural adhesiolysis and SCS can be effective in the long term for controlling chronic back or leg pain due to FBSS, with recommendation grades A and B, respectively. Epidural injections showed a short-term effect (grade C). The evidence regarding the success of other therapies, including revision surgery, medication, exercise, psychotherapy, intrathecal infusion of opioids, and other types of interventions, was poor or inconclusive.

In a second review which also specifically investigated treatment options for FBSS patients with refractory chronic pain, it was concluded that evidence is weak for medications and reoperation, but strong (Level I-II) for active exercise, and some interventional procedures, such as epidural adhesiolysis and SCS [5]. In summary, in both reviews, the strongest evidence for a prolonged effect was obtained for epidural adhesiolysis and SCS, even though the evidence on the efficacy, effectiveness, safety, and cost-effectiveness was found to be insufficient of epidural adhesiolysis for treating FBSS in a recently published systematic review specifically investigating this treatment option [104]. All reviews underscore the need for further research and development of better and longer-term therapeutic options for FBSS patients.

Among the interventional techniques, SCS has been proven to be a very safe and effective therapy in the long term for a variety of chronic pain conditions, and therefore, its use earlier in the treatment algorithm for several of these conditions, including FBSS, has been advocated [69, 105–107]. With further strengthening of the evidence-based support for a sustained long-term efficacy of SCS, this minimally invasive treatment modality may deserve to be put among Level Two treatment options in the FBSS care pathway that has been outlined by the CBLP Network [5, 34, 69, 108].

## 5. Conclusions

Failed back surgery syndrome results from a cascade of medical and surgical events that have led to and left the patient with chronic back and radicular pain. This pain often remains refractory to sporadic (and usually not well-planned) management strategies for a considerable proportion of these patients, highlighting the need for a global, multidisciplinary-based approach. A clear and concise, standardized care pathway comprising recommendations for assessment, treatment, and outcome evaluation using an MDT approach would be an important resource for specialists and nonspecialists who manage patients with FBSS. A comprehensive reference FBSS care pathway has the potential to improve decision-making, reduce variation in practice, and optimize treatment outcomes for this often hard-to-treat condition.

## Figures and Tables

**Table 1:** The therapeutic focus and importance of a multidisciplinary team and the number of experts consulted in the development of each care pathway.

Manuscript identification	Therapeutic focus	Emphasis on MDT	Number (*N*) and spectrum of experts consulted
Avellanal et al. [24]	Epiduroscopy as a diagnostic and therapeutic tool in FBSS	Yes	*N* = 4 Wide
	Psychological and medical management excluded		

Chan and Peng [25]	All considered	Yes	*N* = 2 Narrow

Desai et al. [12]	Medical, rehabilitative, and behavioral treatment	Related to medical, rehabilitative, and behavioral treatment only	*N* = 5 Wide

Durand et al. [20]	Medical management	Discussed in relation to cognitive or behavioral disorders only	*N* = 3 Narrow

Ganty and Sharma [26]	Neuromodulation	Yes	*N* = 2 Narrow

Van Buyten and Linderoth [2]	Neuromodulation	None	*N* = 5 Wide
	Conservative management was not discussed		
	Authors' comment concerning historical algorithms: “several algorithms for the treatment of FBSS that focus largely on diagnosis and possible orthopaedic and neurosurgical interventions have been published; however, the place of SCS in these algorithms has remained unclear”		
